# Perioperative outcome of unilateral versus bilateral inguinal hernia repairs in TAPP technique: analysis of 15,176 cases from the Herniamed Registry

**DOI:** 10.1007/s00464-015-4146-5

**Published:** 2015-03-19

**Authors:** D. A. Jacob, J. A. Hackl, R. Bittner, B. Kraft, F. Köckerling

**Affiliations:** 1 Department of Surgery and Center for Minimally Invasive Surgery, Academic Teaching Hospital of Charité Medical School, Vivantes Hospital, Neue Bergstraße 6, 13585 Berlin, Germany; 2 Hernia Center, Winghofer Medicum, Winghofer Straße 42, 72108 Rottenburg am Neckar, Germany; 3 Department of General and Visceral Surgery and Center for Minimally Invasive Surgery, Diakonie Hospital, Rosenbergstraße 38, 70176 Stuttgart, Germany

**Keywords:** Reoperation, Inguinal hernia, TAPP, TEP, Perioperative complications, Prophylactic repair

## Abstract

**Introduction:**

Following repair of a unilateral inguinal hernia, there is a risk of 1 % per year of onset of an inguinal hernia on the other side. Comparison of bilateral with unilateral TAPP operation in a high-volume center found that morbidity and reoperation rates were only marginally higher for bilateral TAPP operation. Some authors are calling for prophylactic operation of the contralateral side.

**Methods:**

Between September 2009 and April 2013, data were entered into the Herniamed Registry on 15,176 patients who had undergone TAPP operation. Of these patients, 10,887 had been operated on because of a unilateral (71.7 %) and 4289 because of a bilateral (28.3 %) inguinal hernia.

**Results:**

A significant difference was noted in the rate of postoperative complications occurring within 30 days, which was 4.9 % for bilateral compared with 3.9 % for unilateral inguinal hernia (*p* = 0.009). The postoperative complications necessitated reoperation in 0.9 % of patients after unilateral and in 1.9 % of patients after bilateral inguinal hernia repair, thus attesting to the significantly higher risk presented by bilateral inguinal hernia repair (*p* = <0.001).Multivariate analysis confirmed the highly significant influence of bilateral TAPP on increased reoperation rates due to complications (*p* > 0.0001). The odds ratio was 2.13 (95 % CI 1.58–2.86). Comparison of the results from a high-volume center with those from the Herniamed Registry showed that perioperative complication rates were markedly higher.

**Conclusion:**

Perioperative outcome of bilateral TAPP operation demonstrates significantly worse postoperative complication and reoperation rates compared with unilateral TAPP. Likewise, the results were markedly unfavorable compared with those of a high-volume center. If a bilateral hernia repair should be attempted in those patients with only a unilateral hernia, these data give the surgeon more information on how to better prepare a patient and obtain consent preoperatively.

The scientific evidence for laparoscopic/endoscopic repair of bilateral inguinal hernia has been classified as low in the Comparative Effectiveness Review Number 70 of the Agency for Healthcare Research and Quality [1]. Two prospective randomized trials [2, 3] with a total of 114 randomized patients demonstrated that laparoscopic compared with the open technique was associated with significantly less pain, significantly less need for analgesics and significantly earlier resumption of work activities.

A large case series of 2880 bilateral TAPP operations from a high-volume center then revealed that morbidity and reoperation rates were only marginally higher compared with 7240 unilateral TAPP operations [4].

On that scientific basis, laparoscopic/endoscopic repair of bilateral inguinal hernia was recommended by the European Hernia Society [5], the International Endohernia Society [6], European Association of Endoscopic Surgery [7] and the Royal College of Surgeons of England (RCS—Commissioning guide: groin hernias 2013) [8].

When the laparoscopic technique is used to repair a clinically diagnosed unilateral inguinal hernia, it is possible to also explore the contralateral side. In 10–25 % of cases, an asymptomatic, preoperatively inapparent, occult inguinal hernia is identified on the other side [9, 10]. A prospective randomized trial demonstrated that a significant proportion of incidental defects will progress to a symptomatic hernia if left untreated [9]. Accordingly, contralateral occult inguinal hernia found at the time of laparoscopic transabdominal preperitoneal patchplasty (TAPP) repair should also be repaired [11]. The proportion of bilateral inguinal hernias in large clinical series repaired in TAPP technique was 28.5 % [4]. A similar proportion of 28.5 % bilateral inguinal hernias is given in the Herniamed Registry [12] for inguinal hernias repaired using a laparoscopic/endoscopic technique.

Furthermore, studies have demonstrated that following repair of a unilateral inguinal hernia, the likelihood of onset of an inguinal hernia on the contralateral side had to be anticipated in around 1 % of cases for each year of follow-up [13, 14].

In view of the favorable outcome of bilateral repair and the persistent risk of new onset of an inguinal hernia on the other side, which is set at 1 % per year following unilateral repair of inguinal hernia, the merits of prophylactic repair of a healthy groin are discussed in the literature. Zendejas et al. [13] speak about the “role for prophylaxis during endoscopic inguinal hernia repair,” and Lal et al. [14] ask “Is unilateral laparoscopic inguinal hernia repair a job half done?”

The aim of the present analysis of the perioperative findings for 15,176 unilateral and bilateral TAPP operations from the Herniamed Registry was to investigate whether the excellent results obtained for the high-volume center mentioned above could be reproduced on a large scale in several hospitals where surgeons have varying degrees of experience. Based on these multicenter data, it will also be easier to assess whether the perioperative outcome justifies a broad expansion of the indication to prophylactic surgical repair of the healthy side.

## Patients and methods

The Herniamed quality assurance study is a multicenter, internet-based hernia registry into which 358 participating hospitals/surgeons in Germany, Austria and Switzerland (status: April 2013) had entered data prospectively on their patients who had undergone hernia surgery [12]. The analysis now presented here compared the prospectively collected data of all patients who had undergone either unilateral or bilateral repair of inguinal hernia in transabdominal preperitoneal patchplasty (TAPP) between September 2009 and April 2013. Inclusion criteria were minimum age of 16 years, primary inguinal hernia and elective unilateral or bilateral TAPP operation performed under inpatient conditions. In total, 15,176 patients were enrolled. Of these patients, 10,887 had a unilateral (71.7 %) and 4,289 (28.3 %) a bilateral inguinal hernia.

The data on the TAPP operations recorded in the Herniamed Registry originated from 181 out of 358 participating institutions. Forty-three centers, each of which had more than 100 operations, accounted for 77.2 % of the procedures. The remaining 138 centers thus supplied data on 22.8 % operations. Data on 50 % of all unilateral and bilateral TAPP operations came from only 15 hospitals.

The demographic and surgery-related parameters included age (years), sex (m/w), ASA classification (I–IV) as well as the proportion of scrotal inguinal hernias and the hernia defect size based on EHS classification (Grade I–III). The target variables were intra- and postoperative complication rates, number of reoperations as well as the duration of operation and length of hospital stay. The categorical data are displayed as absolute and relative frequencies, and continuous variables are displayed as mean, median, standard deviation and ranges. For the bilateral patient group, data on the variables given for both sides operated on were aggregated. For inguinal hernia defects of different sizes, the side with the larger defect is given. Classification as scrotal hernia was based on the presence of at least one scrotal hernia for bilateral inguinal hernia. Intra- and postoperative complications were recorded if a complication presented on at least one side. The same method was used to present details of any reoperation.

All analyses were performed with the software SAS 9.2 (SAS Institute Inc., Cary, NY, USA) and deliberately reviewed to the full level of significance. Each *p* value ≤0.05 thus represents a statistically significant result. To discern differences between the groups in univariate analysis, Fisher’s exact test was used for categorical target variables, and the t test for continuous target variables. For data that did not follow the normal distribution, as in the case of duration of operation and length of stay, the distribution was first transformed with the natural logarithm. To rule out any skewing of data caused by different patient characteristics, the results of univariate analyses were verified through multivariate analyses in which, in addition to laterality, other influence parameters were simultaneously reviewed. To assess influence factors in multivariate analysis, the general linear model was used for continuous variables, and the binary logistic regression model for dichotomous numeric variables. As performed earlier for univariate analysis, the distribution was transformed for continuous variables. Analyses were based on the number of operated patients and were not adjusted to take account of the number of hernias repaired.

## Results

### Univariate analysis

The mean patient age at 55.4 ± 15.7 years for unilateral inguinal hernia was significantly lower than for bilateral hernia at 56.3 ± 14.6 years (*p* = 0.0005). Likewise, the proportion of men at 86.7 % for unilateral inguinal hernia was lower than for bilateral hernia at 92.8 % (*p* = <0.001). Distribution of ASA classification (*p* = 0.551) was similar in both groups, with the majority of patients belonging to class II. For bilateral inguinal hernias, defect sizes of more than 3 cm were found significantly more often, i.e., Grade III based on the EHS classification (*p* = <0.001). The proportion of scrotal hernias in the unilateral group was 3 %, and in the bilateral group 2.7 % (*p* = 0.421). The results for the demographic and surgery-related parameters are given in Table 1.

**Table 1:** Demographic and surgery-related parameters

Demographic parameters		Unilateral	Bilateral	*p* value
Age	Years ± StdDev Range	55.4 ± 15.7 16–98	56.3 ± 14.6 16–94	0.0005
Sex	Male	9441 (86.72 %)	3980 (92.80 %)	<0.001
	Female	1446 (13.28 %)	309 (7.20 %)	
ASA score	I	3831 (35.19 %)	1539 (35.88 %)	0.551
	II	5725 (52.59 %)	2260 (52.69 %)	
	III	1313 (12.06 %)	483 (11.26 %)	
	IV	18 (0.17 %)	7 (0.16 %)	

Surgery-related parameters		Unilateral	Bilateral	*p* value
Hernia type	Scrotal	325 (2.99 %)	117 (2.73 %)	0.421
EHS classification defect size	Grade I (< 1.5 cm)	1852 (17.01 %)	504 (11.75 %)	<0.001
	Grade II (1.5–3 cm)	6901 (63.39 %)	2644 (61.65 %)	
	Grade III (> 3 cm)	2134 (19.60 %)	1141 (26.60 %)	

*StdDev* standard deviation

The mean duration of operation for unilateral inguinal hernias was 52.62 min, and for bilateral hernias 73.99 min (*p* < 0.0001). The mean length of stay was 1.93 days for unilateral and 2.08 days for bilateral inguinal hernia (*p* < 0.0001). Table 2 gives detailed results for duration of operation and length of stay.

**Table 2:** Duration of operation and length of stay

	Mean	StdDev	Min	Max	Median	*p* value
Duration of operation (min)						
Unilateral	52.62	23.58	20.0	274.0	47.0	*p* < 0.0001
Bilateral	73.99	32.13	20.0	300.0	68.0	
Length of stay (days)						
Unilateral	1.93	2.22	1.0	64.0	2.0	*p* < 0.0001
Bilateral	2.08	2.42	1.0	64.0	2.0	

*StdDev* standard deviation

No significant difference was found between the intraoperative complication rates of 1.4 % for unilateral and 1.2 % for bilateral inguinal hernia (*p* = 0.434). However, a significant difference was noted in the rate of postoperative complications occurring within 30 days, which was 4.9 % for bilateral compared with 3.9 % for unilateral inguinal hernia (*p* = 0.009). This can be explained by the trend toward a higher seroma rate (*p* = 0.082) and a significantly higher rate of intestinal obstructions (*p* < 0.001) following bilateral inguinal hernia repair in TAPP technique. The postoperative complications necessitated reoperation in 0.9 % of patients after unilateral and in 1.9 % of patients after bilateral inguinal hernia repair, thus attesting to the significantly higher risk presented by bilateral inguinal hernia repair (*p* = <0.001).

The incidence of general complications was similar after unilateral (1.2 %) and bilateral (1.5 %) TAPP operation (*p* = 0.182). Five deaths occurred after unilateral repair, but not after bilateral operation (*p* = 0.331). Four of the deceased were between 82 and 90 years and had an ASA classification III or IV score. No intra- or postoperative complications were recorded for any of the cases; however, general complications were documented. The results of perioperative complications and reoperations are given in Table 3.

**Table 3:** Complications and reoperations

Univariate analysis	Unilateral	Bilateral	*p* value
Intraoperative	152 (1.40 %)	52 (1.21 %)	0.434
Bleeding	108 (0.99 %)	36 (0.84 %)	0.404
Injuries (total)	77 (0.71 %)	32 (0.75 %)	0.831
Vascular	34 (0.31 %)	14 (0.33 %)	0.873
Bowel	14 (0.13 %)	6 (0.14 %)	0.808
Bladder	15 (0.14 %)	4 (0.09 %)	0.615
Nerve	0	0	1.000
Postoperative	432 (3.97 %)	211 (4.92 %)	0.009
Bleeding	89 (0.82 %)	40 (0.93 %)	0.492
Seroma	333 (3.06 %)	155 (3.61 %)	0.082
Intestinal lesion	4 (0.04 %)	4 (0.04 %)	0.234
Impaired wound healing	10 (0.09 %)	1 (0.02 %)	0.198
Infection	4 (0.04 %)	3 (0.07 %)	0.412
Intestinal obstruction	6 (0.06 %)	14 (0.33 %)	<0.001
General	137 (1.26 %)	66 (1.54 %)	0.182
Exitus letalis	5 (0.05 %)	0	0.331
Reoperations	98 (0.90 %)	84 (1.96 %)	<0.001

### Multivariate analysis

The absence of difference in the intraoperative complication rates between unilateral and bilateral inguinal hernia repair in TAPP technique demonstrated by univariate analysis was confirmed by the results of multivariate analysis (Table 4; *p* = 0.213). The significant difference in the postoperative complication rate to the disadvantage of bilateral repair was also confirmed by multivariate analysis (*p* = 0.038). The odds ratio was 1.20 (95 % CI 1.01; 1.42). Predictive factors for onset of postoperative complications identified in multivariate analysis included, apart from bilateral operation, a linearly increasing hernia defect, presence of a scrotal hernia and advanced patient age (*p* = <0.0001). The factors identified as influencing onset of postoperative seroma were female gender (*p* = 0.04), a linearly increasing hernia defect, presence of a scrotal hernia and advanced patient age (*p* < 0.0001). Secondary bleeding was significantly more common in larger hernia defects (*p* = 0.008) and advanced patient age (*p* = 0.025). Bilateral TAPP inguinal hernia surgery did not have a significant influence on either the seroma rate (*p* = 0.2014) or on secondary bleeding rate (*p* = 0.7272), influencing only onset of postoperative ileus (*p* = <0.0001). The odds ratio for postoperative ileus after bilateral TAPP was 6.89 (95 % CI 2.58; 18.4).

**Table 4:** Multivariate analysis of perioperative complications

Multivariate analysis	Intraoperative complications	Postoperative complications	General complications
Parameter			
Bilateral hernia odds ratio (95 %CI)	–	0.0382 1.20 [1.01; 1.42]	0.1640 1.24 [0.92; 1.67]
Sex	–	0.0711	0.7842
ASA score	–	0.6158	<0.0001
Defect size	–	<0.0001	0.2776
Scrotal hernia	–	<0.0001	0.8210
Age	–	<0.0001	0.0003

Multivariate analysis	Postoperative seroma	Postoperative bleeding	Postoperative intestinal obstruction
Parameter			
Bilateral hernia odds ratio (95 %CI)	0.2014 1.14 [0.93; 1.38]	0.7272 1.07 [0.73; 1.56]	0.0001 6.89 [2.58; 18.4]
Sex	0.0406	0.2668	0.0353
ASA score	0.5016	0.0954	0.7596
Defect size	<0.0001	0.0081	0.0256
Scrotal hernia	<0.0001	0.8531	0.7026
Age	<0.0001	0.0251	0.8125

In addition, female gender (*p* = 0.035) and the size of the hernia defect (*p* = 0.026) had a significant effect on onset of postoperative ileus in multivariate analysis.

Multivariate analysis also confirmed the highly significant influence of bilateral TAPP on increased reoperation rates (*p* > 0.0001). The odds ratio was 2.13 (95 % CI 1.58; 2.86; Table 5). Besides, linearly increasing hernia defect size was identified as predictive factor for increased reoperation rates (*p* = 0.0091).

**Table 5:** Multivariate analysis of reoperations, duration of operation and length of stay

Multivariate analysis		Reoperation	Duration of operation	Length of stay
Parameter	Bilateral hernia odds ratio (95 %CI)	<0.0001 2.13 [1.58; 2.86]	<0.0001	<0.0001
	Sex	0.8175	0.0462	<0.0001
	ASA score	0.4450	0.0005	<0.0001
	Defect size	0.0091	<0.0001	0.0001
	Scrotal hernia	0.0839	<0.0001	<0.0001
	Age	0.9952	0.7597	<0.0001

The nonsignificant influence of bilateral TAPP on onset of general complications was confirmed in multivariate analysis (*p* = 0.164). Onset of general complications was influenced by advanced patient age (*p* = 0.0003), and linearly increasing ASA classification (*p* < 0.0001; Table 4).

The significant influence of bilateral compared with unilateral TAPP on duration of operation was, as expected, also confirmed by multivariate analysis (Table 5; *p* < 0.0001). Additional predictive influence factors for prolonged duration of operation were male gender (*p* = 0.0462), scrotal hernia (*p* = <0.0001), linearly increasing hernia defect size (*p* < 0.0001) and the ASA score (*p* < 0.0001).

The significantly longer length of stay for patients who had undergone bilateral TAPP operation was also confirmed by multivariate analysis (*p* = <0.0001; Table 5). Other factors influencing the length of stay were female gender (*p* < 0.0001), higher ASA score (*p* < 0.0001), larger hernia defect (*p* < 0.0001) and presence of scrotal hernia (*p* < 0.0001).

## Discussion

The European Hernia Society [5], International Endohernia Society [6], European Association of Endoscopic Surgery [7] and the Royal College of Surgeons of England (RCS—Commissioning guide: groin hernias 2013) [8] recommend laparoscopic/endoscopic repair for bilateral inguinal hernia. When the laparoscopic technique is used to repair a clinically diagnosed unilateral inguinal hernia, occult inguinal hernias are found in 10–25 % of cases on exploration of the contralateral side. If these asymptomatic occult inguinal hernias are left untreated, a significant proportion of them will progress to a symptomatic hernia [9]. Therefore, contralateral occult inguinal hernia found at the time of TAPP repair should also be repaired [11].

Using that approach, the proportion of bilateral inguinal hernias in the total patient collective repaired using a laparoscopic/endoscopic technique was 28.5 % in the Herniamed Registry [12].

The largest case series of 2880 bilateral inguinal hernia repair operations in TAPP technique from a high-volume center revealed that complication rates were only marginally higher compared with 7240 unilateral TAPP operations [4]. In that study the intraoperative complication rate was 0.36 % after unilateral and 0.49 % after bilateral TAPP repair. The postoperative complication rate was 0.77 % after unilateral and 1.4 % after bilateral inguinal hernia repair in TAPP technique. In that large case series, three cases of postoperative ileus also occurred after bilateral, but none after unilateral, repair. The reoperation rates due to postoperative complications in the high-volume center were 0.5 % for unilateral and 0.86 % for bilateral TAPP. In view of these excellent results from a high-volume center, many authors now ponder the merits of prophylactic operation of the other side when repairing unilateral inguinal hernia, since there is a 1 % per year probability of onset of an inguinal hernia on the, until then, healthy side [13, 14]. If one compares the results from a high-volume center with those from the Herniamed Registry, containing data for several participating hospitals and surgeons, markedly higher perioperative complication rates are seen. The intraoperative complication rate for bilateral TAPP in the Herniamed Registry is 1.2 % compared with 0.48 % in the high-volume center. The postoperative surgical complication rate of 4.92 % recorded in the Herniamed Registry was almost three times that of the high-volume center at 1.4 %. Likewise, the reoperation rate due to surgical complications given in the Herniamed Registry was 1.96 % following bilateral TAPP and was thus more than twice that of the high-volume center at 0.86 %. A comparative analysis of unilateral versus bilateral TEP operations in the Herniamed Registry likewise showed a significantly higher postoperative complication and reoperation rate for bilateral TEP [15].

Comparison of a high-volume center with the multicenter data of a hernia registry helps to realistically assess what results can be achieved outside high-volume centers for bilateral repair of inguinal hernias. There is clear evidence here that compared with a high-volume center, when bilateral TAPP is applied on a broad scale (i.e., outside such high-volume center), a twofold higher intraoperative complication rate and reoperation rate, due to the complications arising, and a threefold higher postoperative surgical complication rate must be expected.

Accordingly, the expansion of the indication to prophylactic surgery embodies a decision that should not be taken lightly. The rationale put forward for such a prophylactic intervention is the 1 % risk per year of onset of an inguinal hernia on the contralateral side. If an experienced endoscopic hernia surgeon attempts bilateral hernia repair in those patients with only a unilateral inguinal hernia, these data give the surgeon more information on how to better prepare patients and obtain their consent preoperatively, thus enabling them to decide on their preferred course of action.

Since the results of unilateral TAPP operation in the high-volume center also are markedly better (intraoperative complications: 0.36 % high-volume center versus 1.40 % Herniamed; postoperative surgical complications: 0.77 % high-volume center versus 3.97 Herniamed; reoperation due to surgical complications: 0.50 % high-volume center versus 0.90 % Herniamed), the situation of certified hernia centers must be considered at this juncture. Apparently, many of surgeons who enter data into Herniamed on their operations are still in the learning curve.

A particularly relevant negative effect on the surgical outcome is exerted by larger defects, the presence of scrotal hernia and older patients, who likewise often have larger defects. Bilateral TAPP repair of a larger, possibly scrotal inguinal hernia on one of the two sides calls for a surgeon whose experience goes well beyond the learning curve. Therefore, bilateral TAPP operations for patients who have a large hernia defect or scrotal hernia on at least one side must be performed by the most experienced surgeon in the team. The technical recommendations for correct conduct of TAPP operation must be implemented here as per the guidelines [6].

In summary, it can be stated that bilateral TAPP compared with unilateral TAPP is associated with a significantly higher postoperative complication and reoperation rate because of these complications. This difference is due essentially to a significantly higher rate of intestinal obstructions. Apart from the bilateral procedure itself, large hernia defects, scrotal hernias and a more advanced age have an unfavorable impact on the perioperative outcome. The Herniamed Registry reveals comparable results also for TEP. Compared with the results obtained for a high-volume center, markedly poorer results were observed in cases where several hospitals and surgeons had participated, thus raising the issue of certified hernia centers. Likewise, the indication for prophylactic operation of a healthy groin should be discussed in a critical light with the patient on the basis of the existing data and performed only by very experienced endoscopic inguinal hernia surgeons.

## Appendix: Herniamed Study Group

### Scientific Board

**Köckerling**, Ferdinand (Chairman); **Berger**, Dieter; **Bittner**, Reinhard; **Fortelny**, René; **Koch**, Andreas; **Kraft,** Barbara; **Kuthe**, Andreas; **Lorenz**, Ralph; **Mayer**, Franz; **Moesta**, Kurt Thomas; **Niebuhr**, Henning; **Peiper**, Christian; **Pross**, Matthias; **Reinpold**, Wolfgang; **Simon**, Thomas; **Stechemesser**, Bernd; **Unger**, Solveig.

### Participants

**Alapatt,** Terence Francis (Frankfurt/Main); **Anders,** Stefan (Berlin); **Anderson**, Jürina (Würzburg); **Arndt**, Anatoli (Elmshorn); **Asperger,** Walter (Halle); **Barkus**; Jörg (Velbert); **Becker**, Matthias (Freital); **Berger,** Dieter (Baden–Baden); **Bittner,** Reinhard (Rottenburg); **Blumberg,** Claus (Lübeck); **Böckmann,** Ulrich (Papenburg); **Böhle**, Arnd Steffen (Bremen); **Böttger,** Thomas Carsten (Fürth); **Borchert,** Erika (Grevenbroich); **Born**, Henry (Leipzig); **Brabender,** Jan (Köln); **Breitenbuch von**, Philipp (Radebeul); **Brüggemann**, Armin (Kassel); **Brütting**, Alfred (Erlangen); **Budzier**, Eckhard (Meldorf); **Burghardt**, Jens (Rüdersdorf); **Carus**, Thomas (Bremen); **Cejnar**, Stephan-Alexander (München); **Chirikov**, Ruslan (Dorsten); **Comman,** Andreas (Bogen); **Crescent**i, Fabio (Verden/Aller); **Dapunt**, Emanuela (Bruneck); **Demmel**, Michael (Arnsberg); **Descloux,** Alexandre (Baden); **Deusch**, Klaus-Peter (Wiesbaden); **Dick**, Marcus (Neumünster); **Dieterich**, Klaus (Ditzingen); **Dietz**, Harald (Landshut); **Dittmann**, Michael (Northeim); **Dornbusch**, Jan (Herzberg/Elster); **Drummer**, Bernhard (Forchheim); **Eckermann**, Oliver (Luckenwalde); **Eckhoff,** Jörn/Hamburg); **Elger**, Karlheinz (Germersheim); **Engelhardt**, Thomas (Erfurt); **Erichsen,** Axel (Friedrichshafen); **Eucker**, Dietmar (Bruderholz); **Fackeldey**, Volker (Kitzingen); **Farke**, Stefan (Delmenhorst); **Faust**, Hendrik (Emden); **Federmann**, Georg (Seehausen); **Feichter**, Albert (Wien); **Fiedler,** Michael (Eisenberg); **Fischer**, Ines (Wiener Neustadt); **Fortelny**, René H. (Wien); **Franczak**, Andreas (Wien); **Franke**, Claus (Düsseldorf); **Frankenberg von**, Moritz (Salem); **Friedhoff**, Klaus (Andernach); **Friedrich,** Jürgen (Essen); **Frings**, Wolfram (Bonn); **Fritsche**, Ralf (Darmstadt); **Frommhold,** Klaus (Coesfeld); **Frunder**, Albrecht (Tübingen); **Fuhrer**, Günther (Reutlingen); **Gassler,** Harald (Villach); **Gerdes**, Martin (Ostercappeln); **Glaubitz**, Martin (Neumünster); **Glutig,** Holger (Meißen); **Gmeiner**, Dietmar (Bad Dürrnberg); **Göring**, Herbert (München); **Grebe**, Werner (Rheda-Wiedenbrück); **Grothe**, Dirk (Melle); **Gürtler**, Thomas (Zürich); **Hache**, Helmer (Löbau); **Hämmerle**, Alexander (Bad Pyrmont); **Haffner**, Eugen (Hamm); **Hain**, Hans-Jürgen (Groß-Umstadt); **Hammans**, Sebastian (Lingen); **Hampe**, Carsten (Garbsen); **Harrer**, Petra (Starnberg); **Heinzmann**, Bernd (Magdeburg); **Helbling**, Christian (Rapperswil); **Hempen**, Hans-Günther (Cloppenburg); **Hermes**, Wolfgang (Weyhe); **Herrgesell**, Holger (Berlin); **Herzing**, Holger Höchstadt); **Hessler**, Christian (Bingen); **Hildebrand**, Christiaan (Langenfeld); **Höferlin**, Andreas (Mainz); **Hoffmann**, Michael (Kassel; **Hofmann**, Eva M. (Frankfurt/Main); **Hopfe**r, Frank (Eggenfelden); **Hornung**, Frederic (Wolfratshausen); **Hügel**, Omar (Hannover); **Hüttemann**, Martin (Oberhausen); **Huhn**, Ulla (Berlin); **Imdahl**, Andreas (Heidenheim); **Jacob**, Dietmar (Bielefeld); **Jenert**, Burghard (Lichtenstein); **Junger**, Marc (München); **Käs**, Stephan (Weiden); **Kahraman,** Orhan (Hamburg); **Kaiser,** Christian (Westerstede); **Kaiser**, Stefan (Kleinmachnow); **Kapischke**, Matthias (Hamburg); **Keck**, Heinrich (Wolfenbüttel); **Keller,** Hans W. (Bonn); **Kienzle**, Ulrich (Karlsruhe); **Kipfmüller**, Brigitte (Köthen); **Kirsch**, Ulrike (Oranienburg); **Klammer**, Frank (Ahlen); **Klatt**, Richard (Hagen); **Klein**, Karl-Hermann (Burbach); **Kleist**, Sven (Berlin); **Klobusicky**, Pavol (Bad Kissingen); **Kneifel**, Thomas (Datteln); **Knoop**, Michael (Frankfurt/Oder); **Knotter**, Bianca (Mannheim); **Koch,** Andreas (Cottbus); **Köckerling,** Ferdinand (Berlin); **Köhler**, Gernot (Linz); **König**, Oliver (Buchholz); **Kornblum**, Hans (Tübingen); **Krämer**, Dirk (Bad Zwischenahn); **Kraft**, Barbara (Stuttgart); **Kreissl**, Peter (Ebersberg); **Krones**, Carsten Johannes (Aachen); **Kruse,** Christinan (Aschaffenburg); **Kube**, Rainer (Cottbus); **Kühlberg**, Thomas (Berlin); **Kuhn,** Roger (Gifhorn); **Kusch,** Eduard (Gütersloh); **Kuthe,** Andreas (Hannover); **Ladra**, Jürgen (Düren); **Lahr-Eigen**, Rolf (Potsdam); **Lainka**, Martin (Wattenscheid); **Lammers**, Bernhard J. (Neuss); **Lancee**, Steffen (Alsfeld); **Larusson**, Hannes Jon (Pinneberg); **Lauschke**, Holger (Duisburg); **Leher,** Markus (Schärding); **Leidl**, Stefan (Waidhofen/Ybbs); **Lenz**, Stefan (Berlin); **Lesch**, Alexander (Kamp-Lintfort); **Lienert**, Mark (Duisburg); **Limberger**, Andreas (Schrobenhausen); **Locher**, Martin (Kiel); **Loghmanieh**, Siawasch (Viersen); **Lorenz**, Ralph (Berlin); **Manger**, Regina (Schwabmünchen); **Maurer**, Stephan (Münster); **Mayer**, Franz (Salzburg); **Menzel**, Ingo (Weimar); **Meurer**, Kirsten (Bochum); **Meyer**, Moritz (Ahaus); **Mittenzwey**, Hans-Joachim (Berlin); **Mörder-Köttgen**, Anja (Freiburg); **Moesta**, Kurt Thomas (Hannover); Moldenhauer, Ingolf (Braunschweig); **Morkramer**, Rolf (Xanten); **Mosa**, Tawfik (Merseburg); **Müller**, Hannes (Schlanders); **Münzberg**, Gregor (Berlin); **Mussack,** Thomas (St. Gallen); **Neumann**, Jürgen (Haan); **Niebuhr,** Henning (Hamburg); **Nölling**, Anke (Burbach); **Nostitz**, Friedrich Zoltán (Mühlhausen); **Obermaier**, Straubing); **Öz-Schmidt**, Meryem (Hanau); **Oldorf**, Peter (Usingen); **Pawelzik**, Marek (Hamburg); **Peiper**, Christian (Hamm); **Pertl**, Alexander (Spittal/Drau); **Philipp**, Mark (Rostock); **Pizzera**, Christian (Graz); **Pöllath**, Martin (Sulzbach-Rosenberg); **Possin**, Ulrich (Laatzen); **Prenzel**, Klaus (Bad Neuenahr-Ahrweiler); **Pröve**, Florian (Goslar); **Pronnet**, Thomas (Fürstenfeldbruck); **Pross**, Matthias (Berlin); **Puff**, Johannes (Dinkelsbühl); **Rabl**, Anton (Passau); **Rapp**, Martin (Neunkirchen); **Reinpold,** Wolfgang (Hamburg); **Reuter**, Christoph (Quakenbrück); **Richter,** Jörg (Winnenden); **Riemann**, Kerstin (Alzenau-Wasserlos); **Roehr**, Thomas (Rödental); **Roncossek**, Bremerhaven); **Roth** Hartmut (Nürnberg); **Sardoschau**, Nihad (Saarbrücken); **Sauer**, Gottfried (Rüsselsheim); **Sauer**, Jörg (Arnsberg); **Seekamp**, Axel (Freiburg); **Seelig**, Matthias (Bad Soden); **Seltmann,** Cornelia (Hachenburg); **Senkal,** Metin (Witten); **Shamiyeh**, Andreas (Linz); **Sievers,** Dörte (Hamburg); **Silbernik**, Daniel (Bonn); **Simon**, Thomas (Sinsheim); **Sinn**, Daniel (Olpe); **Sinning**, Frank (Nürnberg); **Smaxwil**, Constatin Aurel (Stuttgart); **Schabel**, Volker (Kirchheim/Teck); **Schadd**, Peter (Euskirchen); **Schattenhofer**, Thomas (Vilshofen); **Scheidbach**, Hubert (Neustadt/Saale); **Schelp**, Lothar (Wuppertal); **Scherf**, Alexander (Pforzheim); **Scheyer**, Mathias (Bludenz); **Schimmelpenning,** Hendrik (Neustadt in Holstein); **Schinkel**, Svenja (Kempten); **Schmid**, Michael (Gera); **Schmid,** Thomas (Innsbruck); **Schmidt**, Sven-Christian (Berlin); **Schmidt,** Ulf (Mechernich); **Schmitz**, Heiner (Jena); **Schoenen**, Detlef (Schwandorf); **Schreiber,** Lutz-Dieter (Bad Langensalza); **Schrittwieser**, Rudolf/Bruck an der Mur); **Schroll**, Andreas (München); **Schultz**, Christian (Bremen-Lesum); **Schultz**, Harald (Landstuhl); **Schulze**, Frank P. Mülheim an der Ruhr); **Schumacher**, Franz-Josef (Oberhausen); **Schwab**, Robert (Koblenz); **Schwandner**, Thilo (Lich); **Schwarz**, Jochen Günter (Rottenburg); **Spangenberger**, Wolfgang (Bergisch-Gladbach); **Sperling**, Peter (Montabaur); **Staade**, Katja (Düsseldorf); **Staib**, Ludger (Esslingen); **Stamm**, Ingrid (Heppenheim); **Stark**, Wolfgang (Roth); **Stechemesser,** Bernd (Köln); **Steinhilper**, Uz (München); **Stern**, Oliver (Hamburg); **Stolte**, Thomas (Mannheim); **Stopinski,** Jürgen (Schwalmstadt); **Stubbe**, Hendrik (Güstrow/); **Stülzebach**, Carsten (Friedrichroda); **Tepel,** Jürgen (Osnabrück); **Terzić**, Alexander (Wildeshausen); **Teske,** Ulrich (Essen); **Thews**, Andreas (Schönebeck); **Tillenburg**, Wolfgang (Marktheidenfeld); **Train**, Stefan H. (Gronau); **Trauzettel**, Uwe (Plettenberg); **Ulcar**, Heimo (Schwarzach im Pongau); **Unger**, Solveig (Chemnitz); **Vogel**, Ulrike (Berlin); **Voit**, Gerhard (Fürth); **Volkers**, Hans-Uwe (Norden); **Vossough**, Alexander (Neuss); **Wallasch**, Andreas (Menden); **Wallner**, Axel (Lüdinghausen); **Warscher,** Manfred (Lienz); **Warwas**, Markus (Bonn); **Weber**, Jörg (Köln); **Weiß**, Johannes (Schwetzingen); **Weißenbach**, Peter (Neunkirchen); **Werner**, Uwe (Lübbecke-Rahden); **Wessel,** Ina (Duisburg); **Wiesner**, Ingo (Halle); **Wolf**, Claudio (Neuwied); **Yildirim**, Selcuk (Berlin); **Zarras**, Konstantinos (Düsseldorf); **Zeller**, Johannes (Waldshut-Tiengen); **Zhorzel**, Sven (Agatharied); **Zuz**, Gerhard (Leipzig).
